# Bedtime smart device usage and accelerometer-measured sleep outcomes in children and adolescents

**DOI:** 10.1007/s11325-021-02377-1

**Published:** 2021-04-30

**Authors:** Paul H. Lee, Andy C. Y. Tse, Teris Cheung, C. W. Do, Grace P. Y. Szeto, Billy C. L. So, Regina L. T. Lee

**Affiliations:** 1 School of Nursing, Hong Kong Polytechnic University, Hung Hom, Hong Kong, China; 2 Department of Health Sciences, George Davies Centre, University of Leicester, University Road, Leicester, LE1 7RH UK; 3 Department of Health and Physical Education, Education University of Hong Kong, Ting Kok, Hong Kong, China; 4 School of Optometry, Hong Kong Polytechnic University, Hung Hom, Hong Kong, China; 5 School of Medical and Health Sciences, Tung Wah College, Kings Park, Hong Kong, China; 6 Department of Rehabilitation Sciences, Hong Kong Polytechnic University, Hung Hom, Hong Kong, China; 7 School of Nursing and Midwifery, University of Newcastle, Callaghan, Australia

**Keywords:** Actigraphy, Cross-sectional, Hong Kong, Smartphone, Youth

## Abstract

**Purpose:**

We analyzed the association between bedtime smart device usage habits and accelerometer-measured sleep outcomes (total sleeping time, sleep efficiency, and wake after sleep onset) in Hong Kong children and adolescents aged 8–14.

**Methods:**

A total of 467 students in Hong Kong participated in this study from 2016 to 2017. They self-reported their bedtime smart device usage habits. The primary caregiver of each participant was also invited to complete a self-administered questionnaire about the family’s social-economic status and bedtime smart device usage habits. An ActiGraph GT3X accelerometer was used to assess participants’ 7-day sleep outcomes.

**Results:**

The mean age of the participants was 10.3 (SD 1.9), and 54% were girls. Among the participants, 27% (*n* = 139) used a smart device before sleep, and 33% (*n* = 170) kept the smart device on before sleep. In total, 27% (*n* = 128) placed the smart device within reach before sleep, 23% (*n* = 107) would wake up when notifications were received, and 25% (*n* = 117) immediately checked the device after being awakened by a notification. Multiple regression controlling for age, sex, socio-economic status, and other confounders showed that those who woke up after receiving a notification had a statistically longer sleeping time (19.7 min, 95% CI: 0.3, 39.1, *p* = 0.046), lower sleep efficiency (− 0.71%, 95% CI − 1.40, − 0.02, *p* = 0.04), and a longer wake after sleep onset (2.6 min, 95% CI: 0.1, 5.1, *p* = 0.045) than those who did not. Nonetheless, all primary caregivers’ bedtime smart device habits were insignificantly associated with all sleep outcomes of their children.

**Conclusion:**

Those who woke up after receiving smart device notifications had lower sleep efficiency and longer wake after sleep onset than those who did not, and they compensated for their sleep loss by lengthening their total sleep time.

**Supplementary information:**

The online version contains supplementary material available at 10.1007/s11325-021-02377-1.

## Introduction

The use of electronic devices, such as television (TV), video games, smartphones, and tablets, is very popular worldwide. Scientific evidence showed that electronic device usage, in particular during bedtime, affects sleep [1]. Much research has investigated the impact of bedtime electronic device usage on sleep, and there are several explanations for this impact. It has been proposed that bedtime use of TV and video games affect sleep because spending more time on them will reduce the available time for sleeping [2], and the blue light emitted from the light-emitting diodes (LEDs) screen may affect circadian rhythms by suppressing the melatonin production of screen users [3]. In recent years, the popularity of smart devices, including smartphones and tablets, has been increasing drastically. In developed countries, most children and adolescents have their own smartphone [4]. Compared with the impact of TV on sleep, it has been argued that bedtime use of a smart device was more disruptive because bedtime smart device usage was more convenient and more common than bedtime TV usage [5]. Smart devices were usually placed near the users during use, and vibrations and ringing functions of the smart device during sleep could interrupt sleep [6, 7].

Several studies have been conducted to examine the impact of the presence and usage before sleep of smartphones and tablets on sleep. Results of these studies showed that the presence of a smart device was associated with less sleeping time and more with sleeping problems among children and adolescents [7–11]. Our secondary data analysis of the 2014 Sleep in America® Poll, conducted by the National Sleep Foundation (NSF), also showed that using a smart device after sleep onset had a strong impact on sleep outcomes among children and adolescents aged 6–17. Those who used electronic communications after sleep onset had shorter sleeping time, were less likely to have good quality of sleep, were more likely to wake up during night, and were more likely to fall asleep in school than those who did not use electronic communications after sleep onset (details of the regression analysis could be found in Supplementary Table S1).

It is worth noting that nearly all existing studies on bedtime smart device usage and sleep relied on self-reported or caregiver-reported sleep outcomes [7–11]. Thus, these measurements of sleep had questionable validity [12], which in turn may have affected the validity of the conclusion drawn from the analysis. Recently, accelerometers, a small electronic device that measures the acceleration as the proxy of movement, have become a popular measurement tool for sleep as their accuracy is comparable with a sleep diary [13]. In this study, we present the results that analyzed the association between bedtime smart device usage habits and accelerometer-measured sleep outcomes (total sleeping time, sleep efficiency, and wake after sleep onset) in Hong Kong children and adolescents aged 8 to 14 years. Because a significant portion of Hong Kong people live in a small apartment (our previous study showed that 86% of Hong Kong primary school students lived in apartments smaller than 55.7 m^2^ [14]), we expected that the bedtime smart device usage habits of the caregivers may also have affected the participants’ sleep outcomes; therefore, these variables were also included in the current data analysis.

## Methods

### Participants

Participants of this study were recruited from five primary schools and four secondary schools in Hong Kong from 2016 to 2017. All schools were public schools and were located in the Kwai Tsing, Tsuen Wan, and Kwun Tong districts of Hong Kong. All students studying primary 3 to 5 in the primary schools (corresponding to grades 3 to 5 in the US education system) and secondary 1 to 3 in the secondary schools (corresponding to grades 7 to 9 in the US education system), aged between 8 and 14 years, were invited to participate. Each participant’s primary caregiver was also invited to complete a self-administered questionnaire. The information sheet was distributed to the participants, and written consent was obtained from one parent of the participant. School teachers collected the signed consent forms and returned them to the research team before the start of the data collection. This study was approved by the Human Subjects Ethics Committee of Hong Kong Polytechnic University (Ref: HSEARS20150722001).

### Measurement

#### Self-administered questionnaire

All participants completed a self-administered questionnaire in the paper-and-pencil format during class with research assistants standing-by to assist. Information on smart device usage (including smartphone, tablets, digital multimedia player, and smart television) concerning number and type of smart devices processed, smart device usage habits, time spent on smart devices in typical school days and holidays/weekends for the whole 24 h, and bedtime smart device usage habits (used smart device before sleep, turned off smart device before sleep, placed smart device within reach before sleep, would wake up if receive smart device notifications or ringing during sleep, and immediately used smart device after waked up by notifications or ringing during sleep) was collected. The original questionnaire in Chinese and its English translation can be found in supplementary materials. Depressive symptoms of the participants were measured using the Center for Epidemiological Studies Depression Scale for Children (CES-DC) [15].

#### Health examination

Height (to the nearest 0.1 cm) and weight (to the nearest 0.1 kg) of the participants were measured by SECA 213 portable stadiometer and Tanita BMI body fat analyzer BC-541 N. All health examinations were conducted by trained research assistants in the halls or on the playgrounds of the participating schools. BMI was computed as weight (kg)/height (m)^2^. Participants were defined as underweight, overweight, and obese according to the international standardized age-gender-specific cutoffs that project the BMI at age 18 to be < 18.5, > 25, and > 30, respectively [16], and the crude BMI was transformed to BMI z-score by the LMS model [17].

#### Caregiver questionnaire

The primary caregiver of each participant (decided among the caregivers) was invited to complete a self-administered questionnaire, which collected basic demographic and socio-economic characteristics of the caregiver (the type of accommodation, primary caregiver’s educational attainment, and monthly household income).

#### Sleep outcomes

An ActiGraph GT3X accelerometer was used to assess participants’ sleep outcomes. Accelerometers are electronic devices that measure the acceleration as a proxy of body movement [18] and have been validated for sleeping time measurement [19, 20]. Within 5 days after the completion of the health examination and questionnaires, participants were instructed to wear the accelerometer on their non-dominant hand for 24 h a day, 7 consecutive days. They were asked to record the removal time in a logbook, for example, temporary removal due to participation in an activity that may damage the accelerometer. Data were collected in 1-min epochs.

### Accelerometer data processing

Peak acceleration in the three axes was extracted from the raw acceleration. The resultant acceleration of the raw acceleration from three axes was summarized by counts per minute (CPM). Non-wearing time was defined by consecutive zero counts for 60 or more minutes [21]. Wearing time in a day was computed by subtracting non-wearing time and sleeping time (determined by the accelerometer) from 24 h. A valid day must include at least 10 h of wearing time excluding sleeping, and only those who provided a minimum of 3 valid days were included in the analysis. The histogram showing the distribution of the non-wearing time is shown in Supplementary Fig. 1. A sensitivity analysis was conducted with all statistical analysis replicated on those who provided at least 1 valid day. All the recorded minutes from accelerometers were classified as either sleep or awake using Sadeh’s algorithm [22]. Sleep onset was defined as the first 15 min of consecutive sleeping minutes, and awakening time was defined as the first 15 min of consecutive wake minutes. Total sleeping time began at sleep onset and ended at awakening [23]. Actual sleeping time was defined as the sleeping time within the total sleeping time. Sleep efficiency was defined as actual sleeping time divided by total sleeping time. Wake after sleep onset (WASO) was defined as the wake time within the total sleeping time. A participant’s sleep outcomes on all valid days were averaged to represent his or her habitual sleep pattern.

Physical activity level was also determined by the accelerometer data. CPM of < 4,514, 4,514–15,044, and ≥ 15,044 will be classified as sedentary, moderate-intensity activity, and vigorous-intensity activity, respectively [24]. Time spent on moderate-to-vigorous physical activity (MVPA) was computed as 2 × vigorous-intensity activity + moderate-intensity activity.

### Statistical analysis

In the original sample, primary 6 students were excluded as the study design required 1-year follow-up and they were going to graduate from the primary schools, which made following-up difficult. Therefore, participants aged 11 were under-represented in our sample. To improve the representativeness of the data analysis results, the sample was weighted according to the Hong Kong By-census 2016 age-gender distribution of aged 8–14 years [25]. Descriptive statistics, including mean, SD, and frequency, were used to summarize the participant characteristics. An independent sample *t*-test was used to analyze the bivariate associations between bedtime smart device usage habits and sleep outcomes. Multiple linear regression was used to analyze the above associations, adjusted for age, sex, time spent on smartphone and tablet, time spent on moderate-to-vigorous physical activity, depressive symptoms, BMI z-score, and caregiver-reported social-economic status. Use of sleep medication (*n* = 13, 2.8%), high blood pressure (*n* = 8, 1.7%), and diabetes (*n* = 3, 0.7%) was rare in our sample; therefore, these variables were not adjusted for. For those who provided at least one day of valid accelerometer data and non-missing responses in bedtime smart device usage habit but with missing values for the aforementioned confounders, the missing values were imputed using multiple imputations. The averages of the regression parameters from 10 imputed datasets by fully conditional specification were reported [26]. To evaluate the sex difference in the associations between bedtime smart device usage habit and sleep outcomes, the fully adjusted regression model was performed in the male and female subsample separately. All data analysis was conducted using IBM SPSS version 25. *p*-value of < 0.05 was considered significant.

## Results

A total of 467 participants who provided at least one day of valid accelerometer data and non-missing responses of bedtime smart device usage habit were included in the current analysis. On average, the participants provided valid accelerometer sleep data for 6.0 nights (SD 1.3). Table 1 shows the demographic characteristics of the participants. The mean age of the participants was 10.3 (SD 1.9), and the sample sex ratio was balanced (54% girls). On school days, the participants spent 3.1 h (SD 2.6) and 0.5 h (SD 1.0) on smartphones and tablets, respectively. On holidays, the time spent on smart devices was nearly doubled. Of the 467 included participants, caregivers of 454 participants (97%) returned the self-administered questionnaire.

**Table 1:** Participant characteristics (*n* = 467). Participants with at least three valid accelerometer days of data were included in the analysis

Variable	Mean	SD
Age	10.3	1.9
Smartphone usage (school day, h/day)	1.6	1.7
Smartphone usage (holiday, h/day)	3.1	2.6
Tablet usage (school day, h/day)	0.5	1.0
Tablet usage (holiday, h/day)	1.1	1.9
Accelerometer-measured MVPA (h/week)	24.5	9.0
CES-D (*n* = 433)	14.8	7.8
Total sleeping time (h/day)	5.8	1.3
Total sleeping time (school day, h/day)	5.6	1.4
Total sleeping time (holiday, h/day)	6.3	1.2
Sleep efficiency (%)	95	3
Sleep efficiency (school day, %)	94	3.0
Sleep efficiency (holiday, %)	98	3
Wake after sleep onset (min/day)	16.7	11.2
Wake after sleep onset (school day, min/day)	19.2	10.4
Wake after sleep onset (holiday, min/day)	10.4	13.0
Variable	Frequency	Percentage
Gender		
Male	213	46
Female	254	54
BMI classification		
Underweight (< 5th percentile)	60	13
Normal (5–85th percentile)	286	61
Overweight (85–95th percentile)	101	22
Obese (> 95th percentile)	20	4
Type of accommodation (caregiver report)		
Public housing	310	69
Home ownership scheme	52	12
Private housing	88	20
Primary caregiver’s level of education (caregiver report)		
No formal education	4	1
Primary	50	11
Secondary	335	79
Tertiary or above	38	9
Monthly housing income (in Hong Kong dollar, caregiver report)		
$0–$9,999	66	15
$10,000–$19,999	175	40
$20,000–$29,999	105	24
$30,000–$39,999	46	10
$40,000–$49,999	26	6
$50,000 +	25	6
Caregiver relationship with the participant (caregiver report)		
Parent	428	95
Grandparent	12	2
Others	14	3
Caregiver’s sex (caregiver report)		
Male	123	27
Female	331	73
Caregiver’s age (caregiver report)		
24 or below	8	2
25–34	53	12
35–44	249	55
45–54	116	26
55–64	27	6
65 or above	3	1

Table 2 shows the participants’ bedtime smart device usage habits and its bivariate association with sleep outcomes. One-fourth of the participants (25%) used a smart device before sleep, and one-third (32%) of them kept the smart device turned on before sleep. About one-fourth of the participants placed the smart device within reach before sleep (27%), would wake up if they receive notifications (23%), and immediately checked the device after being awakened by notification (25%). More than one-third of the primary caregivers (36%) used a smart device before sleep, and nearly half (47%) of them turned them on before sleep. More than one-third of the primary caregivers placed the smart device within reach before sleep (47%), would wake up if they received notifications (34%), and immediately checked the device after being awakened by the notification (38%).

**Table 2:** Association between bedtime smart device usage habit and sleep outcomes

	Total sleeping time (SD)	Sleep efficiency (SD)	Wake after sleep onset (SD)
Participants’ habit			
Used smart device before sleep			
Yes (*n* = 116, 25%)	5 h 57 min/357 min (1 h 12 min/72 min)	95% (3%)	17 min (11 min)
No (*n* = 351, 75%)	6 h 10 min/370 min (1 h 24 min/84 min)	95.5% (3.0%)	17 min (11 min)
*p*-value	0.14	0.24	0.60
Turned off smart device before sleep			
Yes (*n* = 319, 68%)	6 h 12 min/372 min (1 h 25 min/85 min)	96% (3%)	17 min (11 min)
No (*n* = 149, 32%)	5 h 56 min/356 min (1 h 13 min/73 min)	95% (3%)	17 min (11 min)
*p*-value	0.049	0.29	0.73
Placed smart device within reach before sleep			
Yes (*n* = 128, 27%)	5 h 55 min/355 min (1 h 14 min/74 min)	95% (3.%)	17 min (11 min)
No (*n* = 340, 73%)	6 h 11 min/371 min (1 h 24 min/84 min)	95% (3%)	17 min (11 min)
*p*-value	0.054	0.47	0.83
Would wake up if receive smart device notifications or ringing during sleep			
Yes (*n* = 107, 23%)	6 h 18 min/378 min (1 h 22 min/82 min)	95% (3%)	19 min (12 min)
No (*n* = 360, 77%)	6 h 4 min 364 min (1 h 22 min/82 min)	96% (3%)	16 min (11 min)
*p*-value	0.12	0.01	0.006
Immediately used smart device after waked up by notifications or ringing during sleep			
Yes (*n* = 117, 25%)	5 h 58 min/358 min (1 h 14 min/74 min)	95% (3%)	19 min (12 min)
No (*n* = 350, 75%)	6 h 10 min/370 min (1 h 24 min/84 min)	96% (3%)	16 min (11 min)
*p*-value	0.17	0.002	0.008
Primary caregiver’s habit			
Used smart device before sleep			
Yes (*n* = 170, 36%)	6 h 1 min/361 min (1 h 20 min/80 min)	95% (3%)	16 min (11 min)
No (*n* = 297, 64%)	6 h 10 min/370 min (1 h 23 min/83 min)	95% (3%)	17 min (11 min)
*p*-value	0.28	0.94	0.69
Turned off smart device before sleep			
Yes (*n* = 253, 54%)	6 h 11 min/371 min (1 h 24 min/84 min)	96% (3%)	16 min (11 min)
No (*n* = 214, 46%)	6 h 2 min/362 min (1 h 20 min/80 min)	95% (3%)	17 min (12 min)
*p*-value	0.27	0.20	0.15
Placed smart device within reach before sleep			
Yes (*n* = 219, 47%)	6 h 3 min/363 min (1 h 22 min/82 min)	95% (3%)	17 min (12 min)
No (*n* = 248, 53%)	6 h 10 min/370 min (1 h 22 min/82 min)	95% (3%)	17 min (11 min)
*p*-value	0.36	0.97	0.92
Would wake up if receive smart device notifications or ringing during sleep			
Yes (*n* = 158, 34%)	6 h 11 min/361 min (1 h 22 min/82 min)	95% (3%)	17 min (12 min)
No (*n* = 309, 66%)	6 h 3 min/363 min (1 h 22 min/82 min)	95% (3%)	16 min (11 min)
*p*-value	0.32	0.56	0.34
Immediately used smart device after waked up by notifications or ringing during sleep			
Yes (*n* = 175, 38%)	6 h 12 min/372 min (1 h 22 min/82 min)	95% (3%)	17 min (12 min)
No (*n* = 292, 63%)	6 h 4 min/364 min (1 h 22 min/82 min)	95% (3%)	16 min (11 min)
*p*-value	0.32	0.56	0.34

All comparisons conducted by independent sample *t*-test

Table 3 shows the adjusted association between bedtime smart device usage habits and sleep outcomes. Those who woke up if they received smart device notifications had statistically longer sleeping time (19.7 min, 95% CI: 0.3, 39.1, *p* = 0.0046), lower sleep efficiency (− 0.71%, 95% CI − 1.40, − 0.02, *p* = 0.04), and longer wake after sleep onset (2.6 min, 95% CI: 0.1, 5.1, *p* = 0.045) than those who did not. All other habits, including whether the participants used a smart device before sleep, turned off the smart device before sleep, placed it within reach before sleep, and used it during sleep had no association with any sleep outcomes. Participants with a primary caregiver who turned off their smart devices before sleep has shorter wake after sleep onset (2.1 min, 95% CI: 0.1, 4.2, *p* = 0.04). All other primary caregivers’ habits were insignificantly associated with all sleep outcomes.

**Table 3:** Regression coefficients of bedtime smart device usage habit on sleep outcomes

	Total sleeping time (min)		Sleep efficiency (%)		Wake after sleep onset (min)	
Model 1	Beta (95% CI)	*p*-value	Beta (95% CI)	*p*-value	Beta (95% CI)	*p*-value
Participants’ habit						
Used smart device before sleep	− 3.3 (− 22.4, 15.7)	0.73	− 0.21 (− 0.93, 0.50)	0.56	0.41 (− 2.20, 3.03)	0.76
Turned off smart device before sleep	3.0 (− 13.9, 19.9)	0.73	0.30 (− 0.34, 0.94)	0.29	− 0.75 (− 3.08, 1.58)	0.53
Placed smart device within reach before sleep	− 9.8 (− 28.6, 9.0)	0.31	0.20 (− 0.50, 0.91)	0.64	− 0.79 (− 3.38, 1.79)	0.55
Would wake up if receive smart device notifications or ringing during sleep	22.4 (3.0, 41.7)	0.02	− 0.75 (− 1.47, − 0.02)	0.04	3.45 (0.79, 6.11)	0.01
Immediately used smart device after waked up by notifications or ringing during sleep	− 8.8 (− 27.8, 10.2)	0.37	− 0.70 (− 1.41, 0.02)	0.055	2.36 (− 0.25, 4.97)	0.08
Primary caregivers’ habit						
Used smart device before sleep	0.01 (− 16.8, 16.8)	0.999	0.20 (− 0.44, 0.83)	0.54	− 0.79 (− 3.11, 1.52)	0.50
Turned off smart device before sleep	4.2 (− 11.3, 19.8)	0.59	0.38 (− 0.21, 0.96)	0.21	− 1.88 (− 4.01, 0.26)	0.09
Placed smart device within reach before sleep	− 1.0 (− 17.8, 15.8)	0.91	0.45 (− 0.39, 0.87)	0.45	− 0.87 (− 3.18, 1.44)	0.46
Would wake up if receive smart device notifications or ringing during sleep	− 13.7 (− 31.7, 4.3)	0.14	− 0.12 (− 0.79, 0.56)	0.74	− 0.43 (− 2.91, 2.05)	0.74
Immediately used smart device after waked up by notifications or ringing during sleep	10.6 (− 6.1, 27.3)	0.22	0.14 (− 0.49, 0.77)	0.66	− 0.02 (− 2.32, 2.28)	0.99
Model 2	Beta (95% CI)	*p*-value	Beta (95% CI)	*p*-value	Beta (95% CI)	*p*-value
Participants’ habit						
Used smart device before sleep	− 1.8 (− 20.7, 17.0)	0.85	− 0.24 (− 0.95, 0.47)	0.51	0.64 (− 1.94, 3.22)	0.63
Turned off smart device before sleep	3.6 (− 12.9, 20.0)	0.67	0.34 (− 0.29, 0.96)	0.29	− 0.87 (− 3.14, 1.39)	0.45
Placed smart device within reach before sleep	− 5.6 (− 24.0, 12.9)	0.55	0.17 (− 0.53, 0.87)	0.64	− 0.41 (− 2.95, 2.12)	0.75
Would wake up if receive smart device notifications or ringing during sleep	22.0 (3.3, 40.7)	0.02	− 0.71 (− 1.41, 0.0001)	0.05005	3.28 (0.71, 5.84)	0.01
Immediately used smart device after waked up by notifications or ringing during sleep	− 8.6 (− 27.1, 9.8)	0.36	− 0.72 (− 1.42, − 0.02)	0.04	2.52 (− 0.01, 5.06)	0.051
Primary caregivers’ habit						
Used smart device before sleep	1.5 (− 14.8, 17.9)	0.85	0.13 (− 0.49, 0.75)	0.68	− 0.43 (− 2.68, 1.82)	0.71
Turned off smart device before sleep	5.3 (− 9.8, 20.5)	0.49	0.45 (− 0.13, 1.02)	0.13	− 2.01 (− 4.09, 0.07)	0.06
Placed smart device within reach before sleep	− 3.1 (− 19.5, 13.4)	0.71	0.31 (− 0.31, 0.93)	0.33	− 1.26 (− 3.51, 1.00)	0.27
Would wake up if receive smart device notifications or ringing during sleep	− 13.7 (− 31.4, 4.0)	0.13	− 0.11 (− 0.78, 0.56)	0.75	− 0.38 (− 2.81, 2.05)	0.76
Immediately used smart device after waked up by notifications or ringing during sleep	11.0 (− 5.4, 27.4)	0.19	0.05 (− 0.57, 0.67)	0.87	0.29 (− 1.96, 2.54)	0.80
Model 3	Beta (95% CI)	*p*-value	Beta (95% CI)	*p*-value	Beta (95% CI)	*p*-value
Participants’ habit						
Used smart device before sleep	− 0.21 (− 19.6, 19.2)	0.98	− 0.25 (− 0.94, 0.44)	0.48	0.77 (− 1.75, 3.30)	0.55
Turned off smart device before sleep	3.7 (− 13.2, 20.6)	0.67	0.34 (− 0.26, 0.95)	0.27	− 0.94 (− 3.15, 1.27)	0.41
Placed smart device within reach before sleep	− 7.4 (− 26.3, 11.5)	0.44	0.16 (− 0.52, 0.83)	0.65	− 0.46 (− 2.93, 2.00)	0.71
Would wake up if receive smart device notifications or ringing during sleep	19.7 (0.3, 39.1)	0.046	− 0.53 (− 1.23, 0.16)	0.13	2.61 (0.09, 5.13)	0.04
Immediately used smart device after waked up by notifications or ringing during sleep	− 7.1 (− 26.4, 12.3)	0.49	− 0.71 (− 1.40, − 0.02)	0.04	2.56 (0.05, 5.08)	0.045
Primary caregivers’ habit						
Used smart device before sleep	1.7 (− 15.1, 18.4)	0.85	0.25 (− 0.35, 0.85)	0.42	− 0.84 (− 3.03, 1.35)	0.45
Turned off smart device before sleep	5.3 (− 10.3, 21.0)	0.51	0.47 (− 0.09, 1.04)	0.10	− 2.12 (− 4.17, − 0.08)	0.04
Placed smart device within reach before sleep	− 1.9 (− 18.7, 15.0)	0.83	0.27 (− 0.33, 0.87)	0.38	− 1.03 (− 3.22, 1.16)	0.36
Would wake up if receive smart device notifications or ringing during sleep	− 12.3 (− 30.5, 5.9)	0.19	− 0.25 (− 0.90, 0.40)	0.45	0.16 (− 2.21, 2.53)	0.89
Immediately used smart device after waked up by notifications or ringing during sleep	11.8 (− 5.0, 28.6)	0.17	0.15 (− 0.45, 0.75)	0.62	− 0.06 (− 2.25, 2.13)	0.96

Model 1: Adjusted for age and sex

Model 2: Adjusted for age, sex, and time spent on smartphone and tablet

Model 3: Adjusted for age, sex, time spent on smartphone and tablet, time spent on moderate-to-vigorous physical activity, depressive symptoms, BMI, and caregiver-reported social-economic status

A total of 519 participants provided at least 1 valid day and the aforementioned analysis had been replicated in this sample. Results of this sensitivity analysis were shown in the supplementary materials (Tables S1 to S3), and they had no essential difference with the results on the main analysis.

Table 4 shows the adjusted association between bedtime smart device usage habits and sleep outcomes among male and female participants, respectively. Among males, all associations between bedtime smart device usage habits and sleep outcomes were insignificant. Among females, those who used immediately used smart device after wake up by notifications or ringing during sleep have a shorter total sleeping time (28.9 min, 95% CI: 4.6, 53.1, *p* = 0.02), those who have a primary caregiver who placed smart device within reach before sleep had higher sleep efficiency (0.81%, 95% CI: 0.06, 1.56, *p* = 0.04) and shorter wake after sleep onset (3.7 min, 95% CI: 0.9, 6.5, *p* = 0.009), and those who have a primary caregiver who used immediately used smart device after wake up by notifications or ringing during sleep have a longer total sleeping time (21.3 min, 95% CI: 0.4, 42.3, *p* = 0.046).

**Table 4:** Regression coefficients of bedtime smart device usage habit on sleep outcomes, stratified by sex

	Total sleeping time (min)		Sleep efficiency (%)		Wake after sleep onset (min)	
Male	Beta (95% CI)	*p*-value	Beta (95% CI)	*p*-value	Beta (95% CI)	*p*-value
Participants’ habit						
Used smart device before sleep	22.9 (− 7.6, 53.3)	0.15	− 0.70 (− 1.19, 1.04)	0.90	1.97 (− 2.00, 5.94)	0.33
Turned off smart device before sleep	− 11.6 (− 38.1, 14.8)	0.35	0.26 (− 0.74, 1.19)	0.60	− 1.22 (− 4.66, 2.22)	0.49
Placed smart device within reach before sleep	− 3.0 (− 32.6, 26.6)	0.94	0.51 (− 0.57, 1.60)	0.35	− 1.47 (− 5.33, 2.39)	0.46
Would wake up if receive smart device notifications or ringing during sleep	13.3 (− 22.2, 48.8)	0.37	− 0.74 (− 2.04, 0.56)	0.26	3.03 (− 1.60, 7.66)	0.20
Immediately used smart device after waked up by notifications or ringing during sleep	20.3 (− 11.1, 51.8)	0.20	− 0.58 (− 1.73, 0.58)	0.33	3.89 (− 0.21, 7.99)	0.06
Primary caregivers’ habit						
Used smart device before sleep	− 18.9 (− 46.5, 8.8)	0.13	0.25 (− 0.76, 1.26)	0.63	− 2.26 (− 5.87, 1.34)	0.22
Turned off smart device before sleep	− 1.7 (− 26.6, 23.2)	0.90	0.33 (− 0.58, 1.24)	0.48	− 1.96 (− 5.21, 1.29)	0.24
Placed smart device within reach before sleep	19.3 (− 7.3, 45.9)	0.15	− 0.20 (− 1.17, 0.78)	0.69	2.06 (− 1.40, 5.53)	0.24
Would wake up if receive smart device notifications or ringing during sleep	− 1.4 (− 30.1, 27.4)	0.84	− 0.37 (− 1.42, 0.68)	0.49	0.72 (− 3.03, 4.47)	0.71
Immediately used smart device after waked up by notifications or ringing during sleep	− 7.1 (− 34.8, 20.6)	0.56	0.03 (− 0.99, 1.04)	0.96	− 0.12 (− 3.72, 3.49)	0.95
Female	Beta (95% CI)	*p*-value	Beta (95% CI)	*p*-value	Beta (95% CI)	*p*-value
Participants’ habit						
Used smart device before sleep	− 4.1 (− 28.9, 20.7)	0.74	− 0.67 (− 1.53, 0.20)	0.13	1.55 (− 1.67, 4.78)	0.35
Turned off smart device before sleep	11.9 (− 10.2, 33.9)	0.29	0.23 (− 0.55, 1.01)	0.56	− 0.26 (− 3.15, 2.63)	0.86
Placed smart device within reach before sleep	− 17.4 (− 41.3, 6.6)	0.16	− 0.10 (− 0.94, 0.74)	0.81	0.01 (− 3.12, 3.14)	0.996
Would wake up if receive smart device notifications or ringing during sleep	17.8 (− 4.5, 40.1)	0.12	− 0.44 (− 1.22, 0.34)	0.27	2.11 (− 0.80, 5.03)	0.15
Immediately used smart device after waked up by notifications or ringing during sleep	− 28.9 (− 53.1, − 4.6)	0.02	− 0.87 (− 1.72, − 0.02)	0.046	2.08 (− 1.07, 5.23)	0.20
Primary caregivers’ habit						
Used smart device before sleep	12.3 (− 8.3, 32.9)	0.24	0.05 (− 0.68, 0.78)	0.89	0.55 (− 2.16, 3.27)	0.69
Turned off smart device before sleep	11.5 (− 8.5, 31.5)	0.26	0.40 (− 0.30, 1.11)	0.26	− 1.54 (− 4.16, 1.08)	0.25
Placed smart device within reach before sleep	− 14.6 (− 35.9, 6.7)	0.18	0.81 (0.06, 1.56)	0.04	− 3.72 (− 6.51, − 0.93)	0.009
Would wake up if receive smart device notifications or ringing during sleep	− 15.5 (− 38.4, 7.4)	0.17	0.03 (− 0.78, 0.83)	0.95	− 0.83 (− 3.83, 2.16)	0.59
Immediately used smart device after waked up by notifications or ringing during sleep	21.3 (0.4, 42.3)	0.046	0.33 (− 0.41, 1.07)	0.38	− 0.43 (− 3.17, 2.32)	0.76

Adjusted for age, sex, time spent on smartphone and tablet, time spent on moderate-to-vigorous physical activity, depressive symptoms, BMI, and caregiver-reported social-economic status

## Discussion

Our results showed that bedtime smart device usage, turning on or off the smart device, and the location of the smart device were not related to any sleep outcomes measured (total sleeping time, sleep efficiency, and wake after sleep onset) in this study, and only those who would wake up after receiving smart device notifications had longer sleep time (20 min), lower sleep efficiency (0.72%), and longer wake after sleep onset (2.6 min) than those who did not. Our results partially agreed with the results of other studies. These differences represented a small Cohen’s *d* effect size (> 0.2) and clinically significant. Nonetheless, it is well-known that determinants of sleep are multi-factorial, and bedtime smart device usage alone has limited explanatory power. Our sensitivity analysis showed that the association between bedtime smart usage habits and sleep outcomes varied by sex. While null associations were found in male participants, parental bedtime smart device usage was associated with better sleep outcomes among female participants, and the underlying reasons are yet to be examined.

Secondary analysis results of the NSF 2014 poll (Supplementary Table S1) were opposite to our results in that children with a caregiver who used electronic communications after the child had gone to sleep had shorter sleeping time (30 to 77 min) than those who did not. The major difference between our study and the NSF 2014 poll (and other existing studies) was in the measurement method of sleep outcomes. Our study used accelerometer-measured sleep outcomes, which were objective, and other existing studies relied on self-reported or parent-reported sleep outcomes, which we believed were of questionable validity and reliability.

There were several implications from the result that those who woke up if receive smart device notifications had longer sleeping time. First, some researchers proposed the displacement effect of smart devices on sleep, that is, spending more time on smart devices reduces the available time on sleeping [2]. However, our results did not support this hypothesis. Instead, our results suggested that bedtime smart device users had reduced sleeping quality and increased wake after sleep onset, and they would compensate for their sleep loss by lengthening their total sleeping time. Second, our results showed that the presence of smart devices within reach did not affect sleep, and sleep was only affected when users were awakened and used them. Our results may imply the blue light effect on melatonin suppression was minimally affecting sleep outcomes, and the disturbance from ringing and vibration outweighed the blue light effect. The above phenomena were also supported by the results from a study among Canadian children, which reported that bedroom access to a phone had the strongest impact on sleep quality, followed by access to a TV and computer, but access to a tablet was not associated with sleep quality [8, 27]. However, neither melatonin nor blue light exposure was measured in this study and our postulations could only be examined in further research.

In our sample, nearly 70% of the participants lived in public housing with an average household size of about 35.4 m^2^ reported in 2018 [28]. In such a crowded environment, we can assume that the usage of smart devices would not only affect the sleep outcomes of the users but also would impact on other household members. In particular, ringing and vibration from one bedroom are very likely to create disturbance to people sleeping in another bedroom. However, our results did not support this postulation. On the contrary, we found that parental bedtime smart device usage habit was not associated with the index child’s sleep outcomes. Several important confounders of this association, including apartment size, whether the bedroom was shared, and the smart device usage habits of other family members, were not measured in this study. Further studies are warranted to investigate such impacts.

The strength of our study was the objective measurement of sleep outcomes, and that socio-economic status data were collected from the caregivers of the participants, providing a valid assessment of the confounding effects. Our results suggested that further research, in particular, properly designed randomized controlled trials that examine the effect of disabling rings and vibrations of all smart devices at night, is warranted. The major limitation of the current study was the cross-sectional nature of the design. Thus, the possibility of a reciprocal association, that is, those having longer sleeping time might use smart devices in the bed to spend their energy [29], could not be eliminated. The items “Would wake up if receive smart device notifications or ringing during sleep” and “Immediately used smart device after waked up by notifications or ringing during sleep” in our questionnaire could reflect a smart device usage habit, but at the same time might reflect other symptoms such as light sleep. Additional limitations include the potential social desirability of the participants, the lack of a sleep diary to complement the accelerometer data, the suitability of the accelerometer MVPA cutoffs, and the validity of self-report smart device usage habit. To conclude, our study showed that being awakened by a smart device during sleep was associated with longer sleep time, and the presence and use of smart devices at bedtime were not associated with sleep outcomes.

## Supplementary Information

Below is the link to the electronic supplementary material.

Supplementary file1 (DOCX 71 KB)

## Data Availability

The anonymous data are available upon request.

## Code availability

The SPSS syntax is available upon request.
