# Supplementary material for: Bedtime smart device usage and accelerometer-measured sleep outcomes in children and adolescents
Source: Sleep Breath. 2021 Apr 30;26(1):477–87. doi: 10.1007/s11325-021-02377-1 (PMC8856998; doi:10.1007/s11325-021-02377-1)
Supplement: Supplementary file 1 — Supplementary file1 (DOCX 71 KB) [file 11325_2021_2377_MOESM1_ESM.docx]

Table S1. Results of National Sleep Foundation 2014 Sleep in America^®^ Poll (n=1,003)

|  |  | Sleep hours (school day, hr) | | Quality of sleep | | Wake up during a typical night | | Fall asleep in school | |
| --- | --- | --- | --- | --- | --- | --- | --- | --- | --- |
|  |  | Mean | SD |  |  |  |  |  |  |
|  |  | 9.0 | 1.5 |  |  |  |  |  |  |
|  |  |  |  |  | Freq (%) |  | Freq (%) |  | Freq (%) |
|  |  |  |  | Poor | 13 (1.3) | Never | 549 (54.7) | Never | 851 (84.8) |
|  |  |  |  | Fair | 87 (8.7) | Once | 366 (36.5) | Rarely | 124 (12.4) |
|  |  |  |  | Good | 475 (47.3) | Twice | 77 (7.7) | Sometimes | 27 (2.6) |
|  |  |  |  | Excellent | 428 (42.6) | Three or above | 11 (1.1) | Often | 2 (0.2) |
| Variable |  | Beta (95% CI) | p-value | Odds ratio (95% CI) | p-value | Odds ratio (95% CI) | p-value | Odds ratio (95% CI) | p-value |
| Gender | Freq (%) |  |  |  |  |  |  |  |  |
| Male | 512 (51.0) | 0.12 (-0.03, 0.27) | 0.12 | 1.12 (0.87, 1.43) | 0.38 | 1.00 (0.78, 1.29) | 0.99 | 1.50 (1.01, 2.21) | 0.04 |
| Female | 491 (49.0) | Ref |  | Ref |  | Ref |  | Ref |  |
|  | Mean (SD) |  |  |  |  |  |  |  |  |
| Age (yr) | 11.3 (3.5) | -0.18 (-0.20, -0.15) | <0.001 | 0.99 (0.95, 1.03) | 0.68 | 0.96 (0.92, 1.01) | 0.08 | 1.15 (1.07, 1.23) | <0.001 |
| Tablet or smartphone in bedroom | Freq (%) |  |  |  |  |  |  |  |  |
| Yes | 285 (28.4) | -0.14 (-0.35, 0.07) | 0.18 | 1.08 (0.78, 1.52) | 0.68 | 1.10 (0.78, 1.54) | 0.60 | 1.17 (0.72, 1.91) | 0.53 |
| No | 718 (71.6) | Ref |  | Ref |  | Ref |  | Ref |  |
| Phone in bedroom | Freq (%) |  |  |  |  |  |  |  |  |
| Yes | 257 (25.6) | 0.22 (-0.05, 0.49) | 0.10 | 1.09 (0.71, 1.69) | 0.69 | 0.60 (0.38, 0.96) | 0.03 | 1.38 (0.75, 2.56) | 0.30 |
| No | 746 (74.4) | Ref |  | Ref |  | Ref |  | Ref |  |
| Turn on tablet or smartphone during sleep | Freq (%) |  |  |  |  |  |  |  |  |
| Yes | 93 (9.3) | -0.18 (-0.50, 0.14) | 0.27 | 2.17 (1.29, 3.65) | 0.004 | 1.12 (0.66, 1.92) | 0.67 | 1.16 (0.58, 2.29) | 0.68 |
| No | 910 (90.7) | Ref |  | Ref |  | Ref |  | Ref |  |
| Turn on phone during sleep | Freq (%) |  |  |  |  |  |  |  |  |
| Yes | 152 (15.2) | 0.23 (-0.09, 0.56) | 0.16 | 1.31 (0.77, 2.24) | 0.32 | 0.75 (0.43, 1.31) | 0.31 | 1.20 (0.60, 2.42) | 0.60 |
| No | 851 (84.8) | Ref |  | Ref |  | Ref |  | Ref |  |
| Use electronic communications after child had gone to sleep in past 7 days |  |  |  |  |  |  |  |  |  |
| Not at all | 862 (85.9) | Ref |  | Ref |  | Ref |  | Ref |  |
| Just one night | 43 (4.3) | -0.50 (-0.90, -0.10) | 0.02 | 0.30 (0.16, 0.56) | <0.001 | 4.52 (2.26, 9.01) | <0.001 | 4.21 (2.08, 8.53) | <0.001 |
| Two or three nights | 59 (5.9) | -0.53 (-0.87, -0.18) | 0.003 | 0.38 (0.21, 0.68) | 0.001 | 4.27 (2.46, 7.43) | <0.001 | 4.97 (2.62, 9.43) | <0.001 |
| Most or all nights | 39 (3.9) | -1.28 (-1.70, -0.86) | <0.001 | 0.13 (0.06, 0.28) | <0.001 | 2.51 (1.26, 5.02) | 0.009 | 5.95 (2.88, 12.31) | <0.001 |

Supplementary Figure 1. Histogram of the accelerometer non-wearing time (=467)


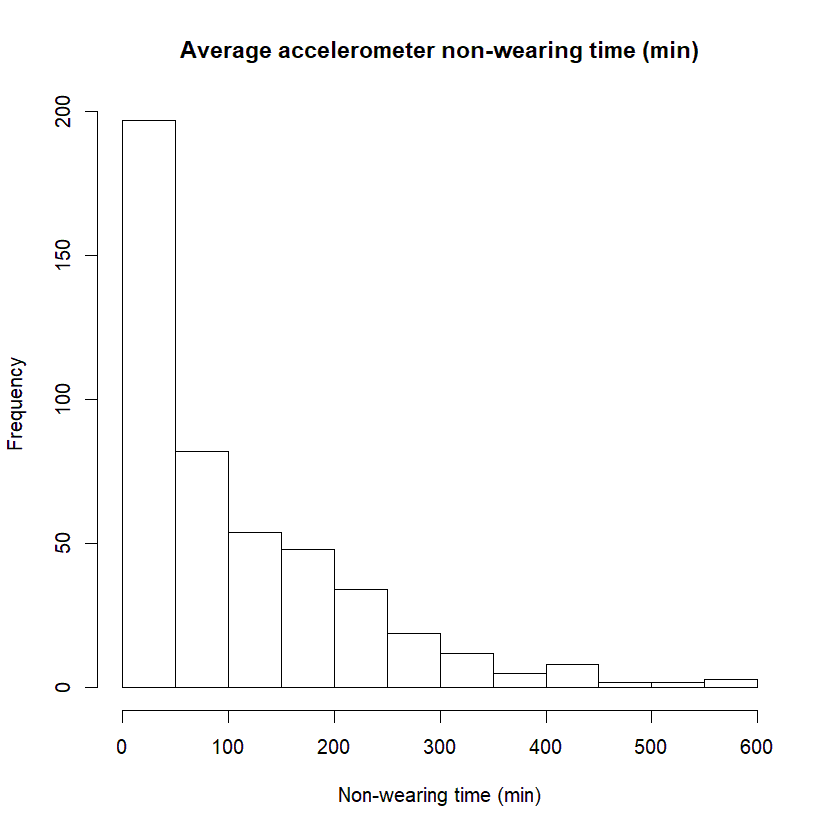


Table S2. Participant characteristics (n=519). Participants with at least one valid acceleromter day of data were included in the analysis.

| Variable | Mean | SD |
| --- | --- | --- |
| Age | 10.44 | 1.96 |
| Smartphone usage (school day, hr/day) | 1.63 | 1.70 |
| Smartphone usage (holiday, hr/day) | 3.21 | 2.72 |
| Tablet usage (school day, hr/day) | 0.50 | 1.03 |
| Tablet usage (holiday, hr/day) | 1.09 | 1.90 |
| Accelerometer-measured MVPA (hr/wk) | 23.55 | 12.82 |
| CES-D (n=476) | 15.09 | 8.11 |
|  |  |  |
| Total sleeping time (hr/day) | 5.8 | 1.4 |
| Total sleeping time (school day, hr/day) | 5.6 | 1.5 |
| Total sleeping time (holiday, hr/day) | 6.3 | 1.2 |
| Sleep efficiency (%) | 95.6 | 3.1 |
| Sleep efficiency (school day, %) | 94.4 | 2.9 |
| Sleep efficiency (holiday, %) | 98.7 | 3.4 |
| Wake after sleep onset (min/day) | 15.7 | 11.2 |
| Wake after sleep onset (school day, min/day) | 18.1 | 10.5 |
| Wake after sleep onset (holiday, min/day) | 9.8 | 13.1 |
| Variable | Frequency | Percentage |
| Gender |  |  |
| Male | 241 | 46.5 |
| Female | 278 | 53.5 |
| BMI classification |  |  |
| Underweight (<5^th^ percentile) | 65 | 12.5 |
| Normal (5^th^ - 85^th^ percentile) | 316 | 60.9 |
| Overweight (85^th^ - 95^th^ percentile) | 116 | 22.4 |
| Obese (>95^th^ percentile) | 22 | 4.2 |
| Type of accommodation (caregiver report) |  |  |
| Public housing | 348 | 69.5 |
| Home ownership scheme | 56 | 11.1 |
| Private housing | 98 | 19.5 |
| Primary caregiver's level of education (caregiver report) |  |  |
| No formal education | 5 | 1.1 |
| Primary | 51 | 10.4 |
| Secondary | 392 | 79.2 |
| Tertiary or above | 46 | 9.3 |
| Monthly housing income (in Hong Kong dollar, caregiver report) |  |  |
| $0-$9,999 | 74 | 15.2 |
| $10,000-$19,999 | 197 | 40.3 |
| $20,000-$29,999 | 113 | 23.0 |
| $30,000-$39,999 | 48 | 9.8 |
| $40,000-$49,999 | 29 | 5.9 |
| $50,000+ | 29 | 5.8 |
| Caregiver relationship with the participant (caregiver report) |  |  |
| Parent | 473 | 94.4 |
| Grandparent | 12 | 2.4 |
| Others | 16 | 3.3 |
| Caregiver’s sex (caregiver report) |  |  |
| Male | 132 | 26.2 |
| Female | 371 | 73.8 |
| Caregiver’s age (caregiver report) |  |  |
| 24 or below | 9 | 1.8 |
| 25-34 | 61 | 12.0 |
| 35-44 | 276 | 54.6 |
| 45-54 | 126 | 25.0 |
| 55-64 | 30 | 5.9 |
| 65 or above | 3 | 0.6 |

Table S3. Association between bedtime smart device usage habit and accelerometer-measured sleep outcomes. Participants with at least one valid acceleromter day of data were included in the analysis.

|  | Total sleeping time (SD) | Sleep efficiency (SD) | Wake after sleep onset (SD) |
| --- | --- | --- | --- |
| Participants’ habit |  |  |  |
| Used smart device before sleep |  |  |  |
| Yes (n=139, 26.8%) | 5 hr 51 min / 351 min (1 hr 21 min / 81 min) | 95.5% (3.2%) | 16 min (11 min) |
| No (n=380, 73.2%) | 6 hr 7 min / 367 min (1 hr 27 min / 87 min) | 95.6% (3.0%) | 16 min (11 min) |
| p-value | 0.06 | 0.71 | 0.75 |
| Turned off smart device before sleep |  |  |  |
| Yes (n=349, 67.2%) | 6 hr 10 min / 370 min (1 hr 29 min / 89 min) | 95.6% (3.0%) | 16 min (11 min) |
| No (n=170, 32.8%) | 5 hr 47 min / 347 min (1 hr 27 min / 87 min) | 95.5% (3.2%) | 16 min (11 min) |
| p-value | 0.004 | 0.64 | 0.82 |
| Placed smart device within reach before sleep |  |  |  |
| Yes (n=141, 27.2%) | 5 hr 50 min / 350 min (1 hr 17 min / 87 min) | 95.5% (3.1%) | 16 min (11 min) |
| No (n=378, 72.1%) | 6 hr 7 min / 367 min (1 hr 29 min / 89 min) | 95.6% (3.1%) | 16 min (11 min) |
| p-value | 0.045 | 0.61 | 0.99 |
| Would wake up if receive smart device notifications or ringing during sleep |  |  |  |
| Yes (n=119, 22.9%) | 6 hr 11 min / 371 min (1 hr 28 min / 88 min) | 94.9% (3.4%) | 18 min (12 min) |
| No (n=400, 77.1%) | 6 hr 0 min / 360 min (1 hr 26 min / 86 min) | 95.8% (2.9%) | 15 min (11 min) |
| p-value | 0.21 | 0.005 | 0.004 |
| Immediately used smart device after waked up by notifications or ringing during sleep |  |  |  |
| Yes (n=142, 26.3%) | 5 hr 50 min / 350 min (1 hr 22 min / 82 min) | 95.0% (3.3%) | 18 min (12 min) |
| No (n=398, 73.7%) | 6 hr 7 min / 367 min (1 hr 27 min / 87 min) | 95.8% (3.0%) | 15 min (11 min) |
| p-value | 0.04 | 0.01 | 0.03 |
| Primary caregiver’s habit |  |  |  |
| Used smart device before sleep |  |  |  |
| Yes (n=190, 36.6%) | 5 hr 55 min / 355 min (1 hr 23 min / 83 min) | 95.7% (3.0%) | 15 min (11 min) |
| No (n=329, 63.4%) | 6 hr 7 min / 367 min (1 hr 27 min / 87 min) | 95.6% (3.1%) | 16 min (11 min) |
| p-value | 0.11 | 0.70 | 0.57 |
| Turned off smart device before sleep |  |  |  |
| Yes (n=281, 54.1%) | 6 hr 9 min / 369 min (1 hr 29 min / 89 min) | 95.7% (3.0%) | 15 min (11 min) |
| No (n=238, 45.9%) | 5 hr 55 min / 355 min (1 hr 22 min / 82 min) | 95.4% (3.1%) | 16 min (12 min) |
| p-value | 0.06 | 0.26 | 0.24 |
| Placed smart device within reach before sleep |  |  |  |
| Yes (n=243, 46.8%) | 5 hr 57 min / 357 min (1 hr 26 min / 86 min) | 95.6% (3.2%) | 16 min (12 min) |
| No (n=276, 53.2%) | 6 hr 8 min / 368 min (1 hr 26 min / 86 min) | 95.6% (3.0%) | 16 min (11 min) |
| p-value | 0.12 | 0.97 | 0.76 |
| Would wake up if receive smart device notifications or ringing during sleep |  |  |  |
| Yes (n=177, 34.1%) | 5 hr 57 min / 357 min (1 hr 26 min / 86 min) | 95.4% (3.5%) | 16 min (12 min) |
| No (n=342, 65.9%) | 6 hr 6 min / 366 min (1 hr 26 min / 86 min) | 95.7% (2.8%) | 15 min (11 min) |
| p-value | 0.21 | 0.25 | 0.33 |
| Immediately used smart device after waked up by notifications or ringing during sleep |  |  |  |
| Yes (n=192, 37.0%) | 6 hr 5 min / 365 min (1 hr 26 min / 86 min) | 95.4% (3.1%) | 17 min (12 min) |
| No (n=327, 63.0%) | 6 hr 1 min / 361 min (1 hr 26 min / 86 min) | 95.7% (3.0%) | 15 min (11 min) |
| p-value | 0.59 | 0.26 | 0.20 |
|  |  |  |  |

All comparison conducted by independent-sample *t*-test.

Table S4. Regression coefficients of bedtime smart device usage habit on sleep outcomes. Participants with at least one valid acceleromter day of data were included in the analysis.

|  | Total sleeping time (min) | | Sleep efficiency (%) | | Wake after sleep onset (min) | |
| --- | --- | --- | --- | --- | --- | --- |
| Model 1 | Beta (95% CI) | p-value | Beta (95% CI) | p-value | Beta (95% CI) | p-value |
| Participants’ habit |  |  |  |  |  |  |
| Used smart device before sleep | -2.9 (-21.2, 15.5) | 0.76 | -0.04 (-0.70, 0.63) | 0.92 | -0.27 (-2.69, 2.16) | 0.83 |
| Turned off smart device before sleep | 6.8 (-9.9, 23.7) | 0.42 | 0.18 (-0.43, 0.79) | 0.56 | -0.45 (-2.66, 1.77) | 0.69 |
| Placed smart device within reach before sleep | -7.5 (-26.0, 11.0) | 0.43 | 0.06 (-0.61, 0.73) | 0.86 | -0.31 (-2.75, 2.13) | 0.81 |
| Would wake up if receive smart device notifications or ringing during sleep | 21.0 (2.0, 40.0) | 0.03 | -0.84 (-1.53, -0.15) | 0.02 | 3.54 (1.03, 6.04) | 0.006 |
| Immediately used smart device after waked up by notifications or ringing during sleep | -12.5 (-30.8, 5.9) | 0.18 | -0.52 (-1.19, 0.14) | 0.12 | 1.77 (-0.66, 4.19) | 0.15 |
| Primary caregivers’ habit |  |  |  |  |  |  |
| Used smart device before sleep | -0.4 (-16.8, 16.1) | 0.97 | 0.20 (-0.40, 0.79) | 0.52 | -0.52 (-2.69, 1.65) | 0.64 |
| Turned off smart device before sleep | 7.6 (-7.9, 23.1) | 0.34 | 0.33 (-0.24, 0.89) | 0.19 | -1.55 (-3.59, 0.50) | 0.14 |
| Placed smart device within reach before sleep | -3.7 (-20.1, 12.8) | 0.66 | 0.26 (-0.34, 0.86) | 0.63 | -1.12 (-3.29, 1.06) | 0.31 |
| Would wake up if receive smart device notifications or ringing during sleep | -14.7 (-32.4, 3.1) | 0.11 | 0.11 (-0.53, 0.76) | 0.73 | -1.08 (-3.42, 1.27) | 0.37 |
| Immediately used smart device after waked up by notifications or ringing during sleep | 10.3 (-6.4, 26.9) | 0.23 | -0.08 (-0.68, 0.53) | 0.81 | 0.53 (-1.67, 2.72) | 0.64 |
| Model 2 | Beta (95% CI) | p-value | Beta (95% CI) | p-value | Beta (95% CI) | p-value |
| Participants’ habit |  |  |  |  |  |  |
| Used smart device before sleep | -0.01 (-18.1, 18.1) | 0.999 | -0.08 (-0.74, 0.58) | 0.82 | 0.08 (-2.31, 2.47) | 0.95 |
| Turned off smart device before sleep | 7.1 (-9.3, 23.4) | 0.40 | 0.21 (-0.39, 0.80) | 0.50 | -0.56 (-2.72, 1.61) | 0.62 |
| Placed smart device within reach before sleep | -3.9 (-22.0, 14.2) | 0.67 | 0.03 (-0.63, 0.69) | 0.93 | -0.04 (-2.43, 2.35) | 0.97 |
| Would wake up if receive smart device notifications or ringing during sleep | 21.1 (2.8, 39.4) | 0.02 | -0.84 (-1.51, -0.17) | 0.01 | 3.52 (1.10, 5.93) | 0.004 |
| Immediately used smart device after waked up by notifications or ringing during sleep | -10.5 (-28.3, 7.4) | 0.25 | -0.56 (-1.21, 0.29) | 0.09 | 2.02 (-0.34, 4.37) | 0.09 |
| Primary caregivers’ habit |  |  |  |  |  |  |
| Used smart device before sleep | 1.6 (-14.7, 17.6) | 0.85 | 0.13 (-0.45, 0.72) | 0.65 | -0.15 (-2.27, 1.97) | 0.89 |
| Turned off smart device before sleep | 8.7 (-6.4, 23.8) | 0.26 | 0.37 (-0.19, 0.92) | 0.19 | -1.62 (-3.62, 0.38) | 0.11 |
| Placed smart device within reach before sleep | -5.7 (-21.8, 10.4) | 0.49 | 0.31 (-0.48, 0.79) | 0.63 | -1.43 (-3.56, 0.69) | 0.19 |
| Would wake up if receive smart device notifications or ringing during sleep | -14.7 (-32.1, 2.6) | 0.18 | -0.21 (-0.80, 0.39) | 0.50 | -1.14 (-3.44, 1.15) | 0.33 |
| Immediately used smart device after waked up by notifications or ringing during sleep | 11.2 (-5.0, 27.4) | 0.18 | -0.33 (-0.85, 0.19) | 0.21 | 0.96 (-1.19, 3.11) | 0.38 |
| Model 3 | Beta (95% CI) | p-value | Beta (95% CI) | p-value | Beta (95% CI) | p-value |
| Participants’ habit |  |  |  |  |  |  |
| Used smart device before sleep | -1.4 (-20.0, 17.3) | 0.89 | -0.28 (-0.93, 0.36) | 0.39 | 0.75 (-1.61, 3.10) | 0.53 |
| Turned off smart device before sleep | 6.4 (-10.4, 23.2) | 0.46 | 0.34 (-0.25, 0.92) | 0.26 | -0.97 (-3.10, 1.15) | 0.37 |
| Placed smart device within reach before sleep | -5.1 (-23.5, 13.3) | 0.59 | 0.12 (-0.52, 0.76) | 0.71 | -0.41 (-2.74, 1.92) | 0.73 |
| Would wake up if receive smart device notifications or ringing during sleep | 19.4 (0.6, 38.3) | 0.04 | -0.64 (-1.29, 0.01) | 0.055 | 2.91 (0.53, 5.28) | 0.02 |
| Immediately used smart device after waked up by notifications or ringing during sleep | -10.1 (-28.7, 8.6) | 0.92 | -0.68 (-1.33, -0.03) | 0.04 | 2.38 (0.03, 4.73) | 0.048 |
| Primary caregivers’ habit |  |  |  |  |  |  |
| Used smart device before sleep | 0.8 (-15.6, 17.3) | 0.29 | 0.29 (-0.29, 0.86) | 0.33 | -0.77 (-2.86, 1.31) | 0.47 |
| Turned off smart device before sleep | 7.4 (-8.2, 23.0) | 0.36 | 0.37 (-0.17, 0.92) | 0.18 | -1.77 (-3.75, 0.20) | 0.08 |
| Placed smart device within reach before sleep | -5.4 (-21.8, 11.0) | 0.52 | 0.29 (-0.28, 0.86) | 0.33 | -1.20 (-3.27, 0.88) | 0.26 |
| Would wake up if receive smart device notifications or ringing during sleep | -9.1 (-27.0, 8.8) | 0.32 | -0.08 (-0.70, 0.55) | 0.81 | -0.25 (-2.51, 2.01) | 0.83 |
| Immediately used smart device after waked up by notifications or ringing during sleep | 12.0 (-4.7, 28.7) | 0.16 | 0.09 (-0.49, 0.67) | 0.77 | 0.04 (-2.07, 2.15) | 0.97 |

Model 1: Adjusted for age and sex

Model2: Adjusted for age, sex, and time spent on smartphone and tablet

Model3: Adjusted for age, sex, time spent on smartphone and tablet, time spent on moderate-to-vigorous physical activity, depressive symptoms, BMI, and caregiver-reported social-economic status

Table S5. Regression coefficients of bedtime smart device usage habit on sleep outcomes, stratified by sex. Participants with at least one valid accelerometer day of data were included in the analysis.

|  | Total sleeping time (min) | | Sleep efficiency (%) | | Wake after sleep onset (min) | |
| --- | --- | --- | --- | --- | --- | --- |
| Male | Beta (95% CI) | p-value | Beta (95% CI) | p-value | Beta (95% CI) | p-value |
| Participants’ habit |  |  |  |  |  |  |
| Used smart device before sleep | 15.0 (-13.7, 43.8) | 0.30 | -0.39 (-1.43, 0.64) | 0.46 | 2.76 (-0.94, 6.47) | 0.19 |
| Turned off smart device before sleep | -7.1 (-33.3, 19.1) | 0.60 | 0.39 (-0.56, 1.34) | 0.42 | -1.60 (-4.98, 1.77) | 0.31 |
| Placed smart device within reach before sleep | 2.3 (-25.9, 30.4) | 0.87 | 0.35 (-0.67, 1.36) | 0.50 | -1.17 (-4.82, 2.48) | 0.58 |
| Would wake up if receive smart device notifications or ringing during sleep | 12.1 (-22.1, 46.4) | 0.49 | -0.78 (-2.02, 0.45) | 0.21 | 2.65 (-1.76, 7.06) | 0.23 |
| Immediately used smart device after waked up by notifications or ringing during sleep | 15.3 (-15.1, 45.7) | 0.33 | -0.61 (-1.70, 0.49) | 0.28 | 3.66 (-0.27, 5719) | 0.06 |
| Primary caregivers’ habit |  |  |  |  |  |  |
| Used smart device before sleep | -16.7 (-43.5, 10.1) | 0.22 | 0.28 (-0.69, 1.25) | 0.57 | -1.86 (-5.33, 1.62) | 0.29 |
| Turned off smart device before sleep | 5.4 (-19.1, 29.8) | 0.67 | 0.16 (-0.72, 1.04) | 0.72 | -1.30 (-4.44, 1.85) | 0.46 |
| Placed smart device within reach before sleep | 12.8 (-12.4, 38.0) | 0.32 | -0.15 (-1.05, 0.76) | 0.75 | 1.49 (-1.76, 4.74) | 0.34 |
| Would wake up if receive smart device notifications or ringing during sleep | 6.4 (-21.8, 34.7) | 0.66 | -0.21 (-1.23, 0.81) | 0.69 | 0.82 (-2.83, 4.48) | 0.67 |
| Immediately used smart device after waked up by notifications or ringing during sleep | -9.0 (-35.8, 17.8) | 0.51 | 0.11 (-0.86, 1.07) | 0.83 | -0.58 (-4.04, 2.88) | 0.72 |
| Female | Beta (95% CI) | p-value | Beta (95% CI) | p-value | Beta (95% CI) | p-value |
| Participants’ habit |  |  |  |  |  |  |
| Used smart device before sleep | -2.6 (-27.0, 21.8) | 0.83 | -0.45 (-1.28, 0.38) | 0.29 | 0.76 (-2.30, 3.83) | 0.63 |
| Turned off smart device before sleep | 12.7 (-9.4, 34.8) | 0.26 | 0.28 (-0.47, 1.04) | 0.46 | -0.46 (-3.25, 2.33) | 0.75 |
| Placed smart device within reach before sleep | -13.8 (-37.7, 10.0) | 0.26 | -0.02 (-0.83, 0.79) | 0.96 | -0.12 (-3.11, 2.87) | 0.94 |
| Would wake up if receive smart device notifications or ringing during sleep | 19.0 (-3.1, 41.1) | 0.09 | -0.63 (-1.39, 0.12) | 0.10 | 2.82 (0.04, 5.61) | 0.047 |
| Immediately used smart device after waked up by notifications or ringing during sleep | -29.2 (-52.9, -5.5) | 0.02 | -0.76 (-1.56, 0.05) | 0.07 | 1.82 (-1.15, 4.78) | 0.23 |
| Primary caregivers’ habit |  |  |  |  |  |  |
| Used smart device before sleep | 8.9 (-11.5, 29.4) | 0.39 | 0.15 (-0.55, 0.85) | 0.68 | 0.29 (-2.30, 2.89) | 0.82 |
| Turned off smart device before sleep | 10.9 (-9.2, 31.1) | 0.29 | 0.41 (-0.27, 1.10) | 0.24 | -1.66 (-4.20, 0.88) | 0.20 |
| Placed smart device within reach before sleep | -18.3 (-39.6, 3.0) | 0.09 | 0.72 (-0.01, 1.44) | 0.054 | -3.46 (-6.14, -0.77) | 0.01 |
| Would wake up if receive smart device notifications or ringing during sleep | -16.2 (-39.0, 6.6) | 0.16 | 0.21 (-0.57, 0.98) | 0.60 | -1.49 (-4.36, 1.38) | 0.31 |
| Immediately used smart device after waked up by notifications or ringing during sleep | 24.1 (3.0, 45.1) | 0.03 | 0.18 (-0.54, 0.89) | 0.63 | 0.11 (-2.54, 2.76) | 0.94 |

Adjusted for age, sex, time spent on smartphone and tablet, time spent on moderate-to-vigorous physical activity, depressive symptoms, BMI, and caregiver-reported social-economic status
